# Genomic characteristics and environmental distributions of the uncultivated Far-T4 phages

**DOI:** 10.3389/fmicb.2015.00199

**Published:** 2015-03-16

**Authors:** Simon Roux, François Enault, Viviane Ravet, Olivier Pereira, Matthew B. Sullivan

**Affiliations:** ^1^Ecology and Evolutionary Biology, University of ArizonaTucson, AZ, USA; ^2^Laboratoire “Microorganismes: Génome et Environnement,” Clermont Université, Université Blaise PascalClermont-Ferrand, France; ^3^Centre National de la Recherche Scientifique, UMR 6023, Laboratoire Microorganismes: Génome et EnvironnementAubière, France

**Keywords:** T4 phages, freshwater ecology, Caudovirales, capsid proteins, viral genomes

## Abstract

Viral metagenomics (viromics) is a tremendous tool to reveal viral taxonomic and functional diversity across ecosystems ranging from the human gut to the world's oceans. As with microbes however, there appear vast swaths of “dark matter” yet to be documented for viruses, even among relatively well-studied viral types. Here, we use viromics to explore the “Far-T4 phages” sequence space, a neighbor clade from the well-studied T4-like phages that was first detected through PCR study in seawater and subsequently identified in freshwater lakes through 454-sequenced viromes. To advance the description of these viruses beyond this single marker gene, we explore Far-T4 genome fragments assembled from two deeply-sequenced freshwater viromes. Single gene phylogenetic trees confirm that the Far-T4 phages are divergent from the T4-like phages, genome fragments reveal largely collinear genome organizations, and both data led to the delineation of five Far-T4 clades. Three-dimensional models of major capsid proteins are consistent with a T4-like structure, and highlight a highly conserved core flanked by variable insertions. Finally, we contextualize these now better characterized Far-T4 phages by re-analyzing 196 previously published viromes. These suggest that Far-T4 are common in freshwater and seawater as only four of 82 aquatic viromes lacked Far-T4-like sequences. Variability in representation across the five newly identified clades suggests clade-specific niche differentiation may be occurring across the different biomes, though the underlying mechanism remains unidentified. While complete genome assembly from complex communities and the lack of host linkage information still bottleneck virus discovery through viromes, these findings exemplify the power of metagenomics approaches to assess the diversity, evolutionary history, and genomic characteristics of novel uncultivated phages.

## Introduction

Viruses are the most abundant biological entities in the biosphere, impact microbial communities structure, alter cellular genomes evolutionary history and indirectly influence major biogeochemical cycles (Breitbart et al., 2007; Suttle, 2007; Rohwer et al., 2009; Hurwitz et al., 2013). Despite these important roles, most viruses in nature (~75–95%) remain uncharacterized in any well-sampled viral community (Brum et al., in press). While new isolates will certainly help with this –e.g., viruses for two of the most abundant marine bacteria (SAR11 and SAR1116) and rare virosphere representatives (infecting *Cellulophaga*) were described only last year (Holmfeldt et al., 2013; Kang et al., 2013; Zhao et al., 2013), rapid means of discovering and characterizing viruses are needed. One approach is to pull viral signals out of microbial genomic datasets, with single-cell amplified genomes (SAGs) likely offering the best hope of obtaining complete viral genomes for uncultivated hosts (e.g., Roux et al., 2014a). Another is to use assembled genomic contigs from viral metagenomes (viromes) to explore the genomic context for novel viral groups (e.g., Emerson et al., 2012; Minot et al., 2012a,b; Dutilh et al., 2014).

Among viruses infecting bacteria (bacteriophages or phages), the T4-like superfamily (*Tevenvirinae*) is one of the most widespread, abundant, and extensively studied group. The *Tevenvirinae* are members of the *Myoviridae* order, tailed bacteriophages with a double-stranded DNA genome, and were first isolated and characterized on *Escherichia coli* (Miller et al., 2003b). Other members of this superfamily were subsequently isolated on *Aeromonas* (Petrov et al., 2010; Kim et al., 2012), *Vibrio* (Miller et al., 2003a), *Prochlorococcus* and *Synechococcus* (Sullivan et al., 2010), and *Pelagibacter* (Zhao et al., 2013).

The abundance of T4 phages in natural communities, largely assessed by marker genes, has been the subject of significant effort since initial PCR-based analyses were implemented in 1998 (Fuller et al., 1998). Subsequent studies, targeting the portal protein (T4 phage gene 20) and major capsid protein (MCP, T4 phage gene 23) genes, ensued across marine (Millard et al., 2004; Filée et al., 2005; Zeidner et al., 2005; Sullivan et al., 2006, 2008; Sharon et al., 2007; Comeau and Krisch, 2008; Goldsmith et al., 2011), and freshwater (Dorigo et al., 2004; Chénard and Suttle, 2008; Butina et al., 2010; Matteson et al., 2011; Hewson et al., 2012) samples. While criticized as a means to quantitatively evaluate T4 phage ecology (Sullivan et al., 2008; Duhaime and Sullivan, 2012; Sullivan, 2015), such marker gene surveys have clearly helped document the diversity of T4 phage marker genes and establish hypotheses about evolutionary history and taxonomy in wild T4 phages. Specifically, the *Tevenvirinae* appear comprised of several subgroups including (i) the “true” T-evens represented by T4 and closely related phages infecting *Enterobacteria* (e.g., T2, T6), (ii) the Pseudo T-evens and Schizo T-evens (including *Aeromonas* and *Vibrio* phages), morphologically distinguishable, and (iii) the more distant Exo T-evens (including cyano- and pelagiphages).

Beyond marker genes, the T4 phage group has also been relatively extensively explored at the whole genome level. A “core-genome” shared across all or most members of the *Tevenvirinae* was defined, representing functions like DNA replication, repair and recombination, virion morphogenesis or control of gene expression (Sullivan et al., 2005, 2010; Petrov et al., 2010). Further, hierarchical “core” gene sets from subsets of these phages and flexible genes sporadically distributed across these genomes suggested means by which T4 phages differentiate to different environments and hosts (Millard et al., 2004; Mann et al., 2005; Weigele et al., 2007; Petrov et al., 2010; Sullivan et al., 2010). The largely similar genome organization and predominantly vertical evolutionary history of core genes hint at robust taxonomic boundaries in this phage group (Ignacio-Espinoza and Sullivan, 2012), and recent exploration of genomic variability in wild T4-like cyanophages confirmed such discrete structure in sequence space and empirically placed limits between populations at about 95% nucleotide identity (Deng et al., 2014).

T4-like phage sequences were also mined from the Global Ocean Sampling (GOS) expedition microbial metagenomic dataset (i.e., the viral signal here originate from actively infected cells captured on filters) to design new degenerate PCR primers which revealed a new T4 phage group—the “Far-T4” phages (Comeau and Krisch, 2008). This clade includes a very peculiar phage: RM378, isolated on the thermophilic bacterium *Rhodothermus marinus* (Hjorleifsdottir et al., 2014). Morphologically, phage RM378 is similar to a T4-like phage (A2 morphology) and encodes a T4-like capsid protein gene, but its genome contains only half of the 38 (core) genes conserved in 26 T4-like phage genomes available for comparative study (Sullivan et al., 2010). Moreover, the RM378 genome lacks a readily identifiable structural or replication module that is discernible among all other *Tevenvirinae*. Far-T4 phage major capsid proteins have since then been detected in marine (Williamson et al., 2012; Hurwitz and Sullivan, 2013) and freshwater (Roux et al., 2012) viromes, but no formal genomic evaluation of the Far-T4 phages is available beyond the reference RM378 genome (Hjorleifsdottir et al., 2014).

Here, to expand our understanding of Far-T4 phages, we assembled genome fragments from two deeply-sequenced freshwater viromes, and used these to evaluate the evolutionary history of the Far-T4 phages and of their major capsid protein, as well as assess their global distribution in freshwater and marine ecosystems using 196 previously published viromes.

## Results

### Detection of Far-T4 contigs

**Figure 1:**
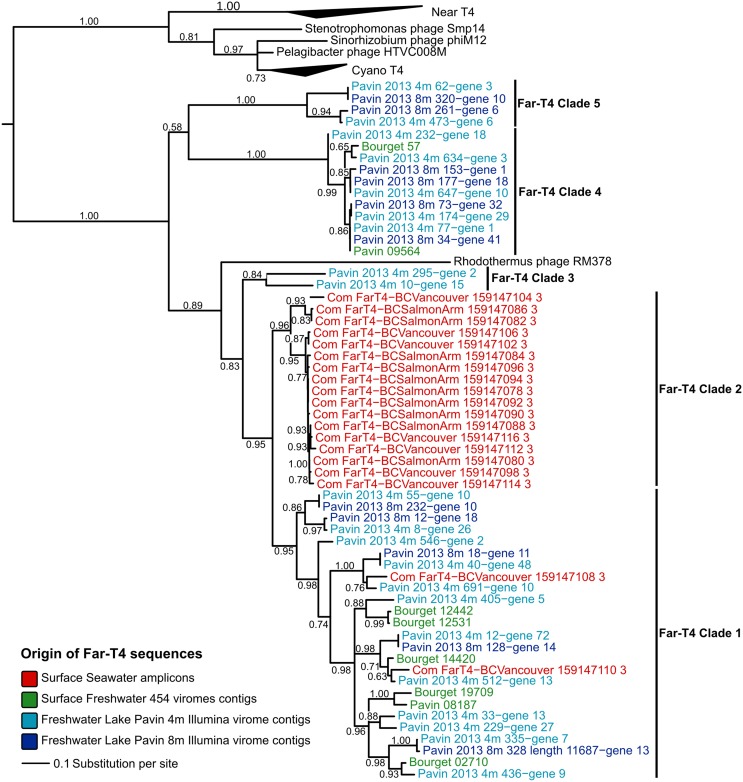
**Phylogenetic tree computed from Gp23 (major capsid protein) multiple alignment**. As this tree includes the PCR sequences used to define the Far-T4 group, the multiple alignment was trimmed to include only positions matching these amplicons. All nodes with bootstraps lower than 50 were collapsed. Far-T4 sequences are color-coded according to their original sample, and reference sequences are in black.

Reads from 2 deeply-sequenced viromes from the Lake Pavin (sampled at 4 and 8 m) and 2 previously published 454 viromes from surface samples of Lakes Pavin and Bourget (Roux et al., 2012) were assembled into genome fragments and searched for *g23* genes. Overall, 32 Far-T4 *g23* genes were detected in the two deeply-sequenced viromes, and eight in the 454 viromes (Table 1). Using these and publicly available sequences, the diversity and structure of the Far-T4 phages was evaluated using a Gp23-based phylogenetic tree (Figure 1). This tree clearly resolves the T4-like phages (“Near-T4”) from the T4-like cyanophages (“Cyano-T4”), along with two recently described *Alphaproteobacteria* phages (infecting SAR11 and *Sinorhizobium*) and the Far-T4 phages.

**Table 1:** **List of Far-T4 contigs assembled from freshwater viromes**.

**Dataset**	**Sequence Id**	**Length**	**Clade**	**Marker genes**	**PhoH**	**Putative host group (CRISPR)**	**Putative host family (tetranucleotide)**
HiSeq Viromes	**Pavin_2013_4m_8**	**105162**	**Far_T4_1**	***g23; g20; g17***	**+**	*Clostridia ?*	*Anaplasmataceae ?*
	**Pavin_2013_4m_10**	**97022**	**Far_T4_3**	***g23; g20; g17***	**+**		
	Pavin_2013_4m_12	86378	Far_T4_1	*g23; g20; g17*	+		
	Pavin_2013_8m_12	59230	Far_T4_1	*g23; g20; g17*	+		
	Pavin_2013_4m_33	55178	Far_T4_1	*g23; g20; g17*	+		
	Pavin_2013_4m_40	51859	Far_T4_1	*g23; g20; g17*	+		*Anaplasmataceae ?*
	Pavin_2013_8m_18	51839	Far_T4_1	*g23; g20; g17*	+		*Anaplasmataceae ?*
	Pavin_2013_4m_55	43868	Far_T4_1	*g23; g20; g17 (partial)*	+		
	**Pavin_2013_4m_62**	**40954**	**Far_T4_5**	***g23***			
	**Pavin_2013_4m_77**	**36429**	**Far_T4_4**	***g23***	**+**		
	Pavin_2013_8m_34	35829	Far_T4_4	*g23*	+		
	Pavin_2013_8m_73	27012	Far_T4_4	*g23*	+		
	Pavin_2013_4m_174	23723	Far_T4_4	*g23*	+		
	Pavin_2013_4m_229	20766	Far_T4_1	*g23*	+		
	Pavin_2013_4m_232	20663	Far_T4_4	*g23; g20; g17*			
	Pavin_2013_8m_128	19577	Far_T4_1	*g23*	+		
	Pavin_2013_8m_153	17698	Far_T4_4	*g23*	+		
	Pavin_2013_4m_295	17576	Far_T4_3	*g23*			
	Pavin_2013_8m_177	16514	Far_T4_4	*g23*	+		
	Pavin_2013_4m_335	16154	Far_T4_1	*g23; g20; g17*	+	*Bacilli ?*	
	Pavin_2013_4m_405	14371	Far_T4_1	*g23*	+		
	Pavin_2013_8m_232	14013	Far_T4_1	*g23; g20; g17 (partial)*	+		
	Pavin_2013_4m_436	13727	Far_T4_1	*g23*	+		
	Pavin_2013_4m_473	13214	Far_T4_5	*g23*			
	Pavin_2013_8m_261	13214	Far_T4_5	*g23*			
	Pavin_2013_4m_512	12683	Far_T4_1	*g23*	+		
	Pavin_2013_4m_546	12156	Far_T4_1	*g23*	+		
	Pavin_2013_8m_320	11847	Far_T4_5	*g23*			
	Pavin_2013_8m_328	11687	Far_T4_1	*g23; g20; g17*		*Bacilli ?*	
	Pavin_2013_4m_634	10875	Far_T4_4	*g23*			
	Pavin_2013_4m_647	10681	Far_T4_4	*g23; g20*			
	Pavin_2013_4m_691	10076	Far_T4_1	*g23; g20*			
454 Viromes	Pavin_2009_08187	5217	Far_T4_1	*g23*			
	Bourget_2009_19709	2282	Far_T4_1	*g23*	+		
	Bourget_2009_14420	3756	Far_T4_1	*g23*	+		
	Bourget_2009_12531	2307	Far_T4_1	*g23*	+		
	Bourget_2009_12442	2049	Far_T4_1	*g23*	+		
	Pavin_2009_09564	1527	Far_T4_4	*g23*			
	Bourget_2009_57	1630	Far_T4_4	*g23*			
	Bourget_2009_02710	2702	Far_T4_1	*g23*			

*Contigs selected as reference for each clade are highlighted in bold*.

These latter sequences form a monophyletic group composed of (i) *Rhodothermus* phage RM378, the only reference Far-T4 genome available (in black), (ii) the PCR-amplified sequences from seawater used to first define the Far-T4 group (in red), (iii) 8 sequences retrieved from 454-sequenced and published freshwater lakes viromes (in green), and (iv) 32 sequences from the 2 viromes analyzed here sampled from Lake Pavin (in light and dark blue). Within this monophyletic Far-T4 group, five clades can be robustly delineated (bootstraps >80%) including (i) a clade of both seawater amplicons and freshwater virome-derived sequences that represents the majority of the sequences (Far-T4 clade 1), (ii) a clade of seawater amplicons only (Far-T4 clade 2), and (iii) three clades composed solely of freshwater virome sequences (Far-T4 clades 3, 4, and 5). Notably, the Gp23 sequence of Rhodothermus marinus phage RM378 is clearly distinct from all other Far-T4 phage group members, so its usefulness as a reference genome for the Far-T4 group may be limited.

Phylogenetic trees computed from two other phylogenetic markers—the portal protein (Gp20) and large terminase subunit (Gp17) – confirmed the robust and well-supported monophyly of the Far-T4 phages and the delineation of clades 1, 3, 4, and 5 (Figure S1, only *g23* amplicons are available for clade 2, which could thus not be detected with another marker gene).

### Insights into Far-T4 gene content and genome organization

**Figure 2:**
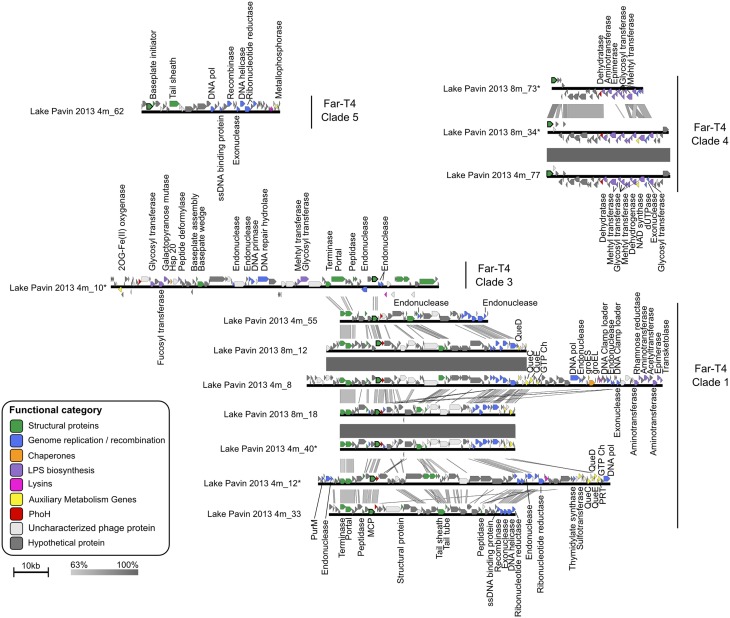
**Comparison of Far-T4 contigs assembled from the Lake Pavin viromes**. The two deeply sequenced viromes from Lake Pavin allowed for the assembly of large genome fragments, which are here compared in terms of gene similarity and order. Genes are color-coded according to their functional annotation, and major capsid genes (g23) are outlined in black. Significant sequence similarity between successive sequences are highlighted by gray squares, shaded according to percentage of amino-acid identity. Predicted proteins with no associated function are classified as “Uncharacterized phage proteins” when at least one similar sequence was detected in a known phage and “Hypothetical protein” otherwise. PurM, Phosphoribosylformylglycinami-dine cyclo-ligase; GTP Ch, GTP cyclohydrolase; DNA Pol, DNA polymerase; PRT, Phosphoribosyl transferase; Hsp, Heat shock protein.

We next used the 12 Far-T4 contigs longer than 25 kb to evaluate the genome content and organization of these new phages. As with all *Tevenvirinae* except RM378, clear preservation of gene order and functional modularity could be delineated (Figure 2). These include structural genes (e.g., portal, terminase, and tail genes) proximal to the major capsid protein (MCP), and replication genes located in a module elsewhere in the genome including DNA polymerases, primases, helicases and exonucleases (Figure 2). Surprisingly, nearby to the structural genes was also a *phoH* gene, which in *E coli* represents a phosphate starvation-induced ATPase (Kim et al., 1993). The *phoH* gene is “core” to marine T4-like cyanophages (Sullivan et al., 2010), has been documented across a wide variety of phages, and used as a marker gene to assess marine phage diversity (Goldsmith et al., 2011), but its function as a phosphate-stress related gene outside of *E. coli* remains controversial (Sullivan et al., 2010). Taken together, the facts that a *phoH* gene occurs in 3 of 4 Far-T4 phage clades for which genome fragments are available (Table 1), is detected near to the MCP for 2 clades (Far-T4 1 and 4), and that trees from PhoH are consistent with the other virion-associated marker genes (Figure S1) suggests strong conservation and likely an important function for this gene in freshwater phages.

Alongside these more typical viral genes, Far-T4 contigs from clade 1 also harbor several Auxiliary Metabolic Genes [AMGs, *sensu* (Breitbart et al., 2007)], notably from the queuosine biosynthesis pathway classifed as class II AMGs (Hurwitz et al., 2015). Queuosine (Que) is a hyper-modified guanosine used in tRNAs specific for four amino acids (Asp, Asn, His, or Tyr) and found across all domains of life (El Yacoubi et al., 2012). This detection of *Que* genes in freshwater Far-T4 complements the recent description of near-complete Que biosynthesis pathway in phages infecting virulent *Streptococcus pneumoniae* strains (Sabri et al., 2011), as well as marine *Cellulophaga baltica* (Holmfeldt et al., 2013), the latter being also detected in in diverse aquatic viromes (55 of 137 screened, Holmfeldt et al., 2013). Taken together, these detections of *Que* genes in phage genomes sampled from such different ecosystems (seawater, freshwater and human lung) suggest a general role in phage cycle for these genes. Interestingly, Sabri et al. (2011) suggested that *Que* genes could act as a feedback signal to control the quantity of phage structural gene transcripts, an hypothesis that would be consistent with the location of these genes in the structural module in Far-T4 contigs.

We next evaluated the diversity and novelty of genes within all Far-T4 contigs. On average, clade 1 genome fragments are the most closely related to database representatives, ~80% of genes have a hit against the NCBI NR database, whereas this frequency is only ~70, 60, and 47% for clades 5, 4, and 3 respectively (Figure S2). Of the novel genes (not hitting anything in databases) genes, 60 to 100% remain conserved within a clade for clades 1, 4, and 5. In contrast, most (80%) of the novel genes in clade three remain unique to each contig and appear not to be conserved within this clade, even though the contigs cover the same genome region including the MCP. This difference in genome content associated with the long distance separating these two sequences on the MCP tree (Figure 1) and the lower bootstrap support for this clade compared to other Far-T4 clades (84% vs. 95–100%) suggest that these sequences may actually represent two neighbor clades rather than one single group.

Despite the fact that these contigs represent incomplete genome fragments of Far-T4 phages, a clustering based on the proportion of genes shared between pairs of contig consistently recovered the clades established using phylogenetic markers (Figure S3, Robinson-Foulds distance between topologies = 30, the same distance between 1000 randomly permuted trees has an average of 51.4, *p*-value < 10^−16^). This indicates that contigs from the same clade indeed share more genes between themselves than with other Far-T4 phages. Accordingly, similarity at the nucleotide level is mostly restricted within each clade and barely detectable between Far-T4 clades (Figure 2). Finally, this comparison at the nucleotide level highlighted identical contigs separately assembled in the 4 and 8 m samples for each clades one and four, suggesting that virome assembly produced consistent results.

### Conservation and evolution patterns of the major capsid protein (MCP)

Given the importance of the major capsid protein (MCP) for evaluating the evolutionary history of viruses (Hendrix, 2002; Bamford et al., 2005; Brüssow, 2009; Abrescia et al., 2012), we next extracted the complete MCP sequences (as opposed to the partial amplicons previously available) from our Far-T4 contigs. The Far-T4 phages MCP was previously noted as “very divergent from the rest of the known sequences” (Comeau and Krisch, 2008), which can be linked to an ancient separation between the Far-T4 and the other T4-like phages, or to a relaxed selection pressure on the MCP in the Far-T4 lineage driven by phage-host coevolution dynamics (Hall et al., 2011).

Broadly speaking, Far-T4 MCP sequences are ~15% longer than their Near-T4 and Cyano-T4 counterparts for all clades (Figure S4). Evolutionarily, the ratio of non-synonymous to synonymous mutations (dN/dS) across the alignment (all T4) is low (0.104 as estimated by PAML; Yang, 2007), which corresponds to a strong stabilizing selection as expected for a functionally important and conserved gene. When allowing for different dN/dS for the Far-T4 phages and the other *Tevenvirinae*, the ratio was only slightly higher in the Far-T4 phages (0.118 vs. 0.093, Table S1). This suggests a strong conservation of the MCP overall, and a sequence divergence between Far-T4 and other T4 phages MCPs likely linked to an ancient separation rather than differences in phage-host interactions.

**Figure 3:**
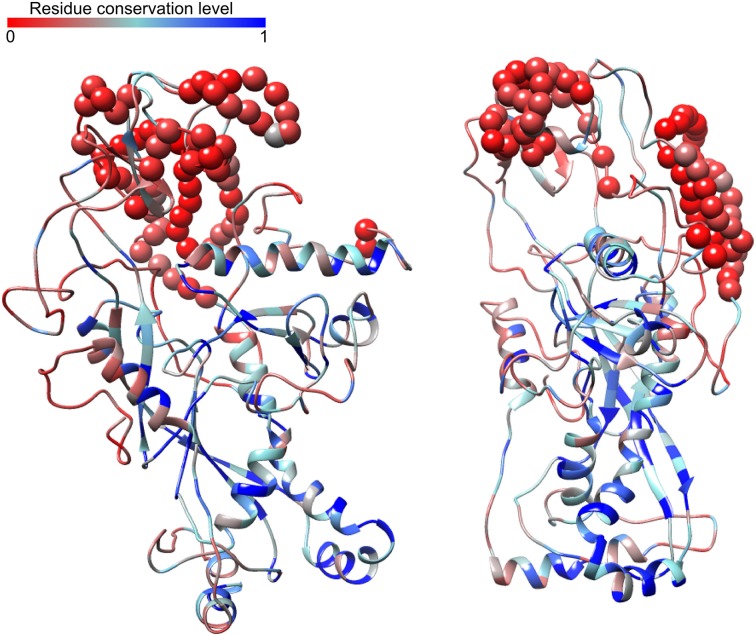
**Predicted structure of the Far-T4 major capsid protein (two views rotated by 90° through the y-axis)**. This model is based on the sequence from contig Pavin_2013_4m_8, and was predicted with I-Tasser based on the structure of the T4 phage vertex protein. Models are colored according to their conservation across T4-like phages, from least (red) to most (blue) conserved. Residues corresponding to insertions in the Far-T4 sequence not present in other T4 phages are highlighted with spheres.

However, 20 out of 302 sites appeared to be under relaxed selection (dN/dS = 1, *p*-value 8.7e-102, Table S1), and corresponded to less-conserved residues between highly conserved regions with predicted secondary structures (Figure S5). To investigate this further, we next built 3D models from our assembled Far-T4 MCP, based on the characterized structure common to the T4 phage MCP and vertex protein. This model suggests the following organization: the N- and C-terminal conserved domains are gathered within a “core” conserved region which includes the predicted secondary structures (blue parts on Figure 3), flanked by more variable and unstructured parts (i.e., no predicted alpha helix or beta strands) on the outside. Similar folding was predicted for the different Far-T4 clades (Figure S6). It is thus tempting to speculate that the conserved and structured parts are responsible for the core virion structure, and that the more variable parts outside are linked to virion-specific decorations as for the known T4-like MCP structure (Comeau and Krisch, 2008).

### Distribution and abundance of Far-T4 in aquatic environments

To evaluate the distribution and relative abundance of Far-T4 phages in nature, we next used our new reference genome fragments for recruitment analysis against 186 publicly available viromes derived from marine (62), freshwater (26), hypersaline (13) or eukaryote-associated (85) samples. To consider a Far-T4 phage as being “present” in a virome, we required at least 100 reads to be recruited per genome fragment (blastp *e*-value < 0.001, score > 50, see Materials and Methods). Overall, Far-T4 phages were detected in 15 freshwater (from López-bueno et al., 2009; Rosario et al., 2009a; Roux et al., 2012; Ge et al., 2013; Tseng et al., 2013) and 37 seawater viromes (from Williamson et al., 2008, 2012; Hurwitz and Sullivan, 2013), or ~60% of all temperate aquatic viromes (i.e., not hypersaline), which included samples from the Pacific, Indian and Atlantic oceans as well as lakes in Europe, Asia, Antarctica and North America (Table S2). All but one of these viromes included sequences similar to every Far-T4 clade (clade five was not detected in the Spring sample from Lake Limnopolar), which suggests that the whole Far-T4 group is relatively widespread among aquatic environments.

**Figure 4:**
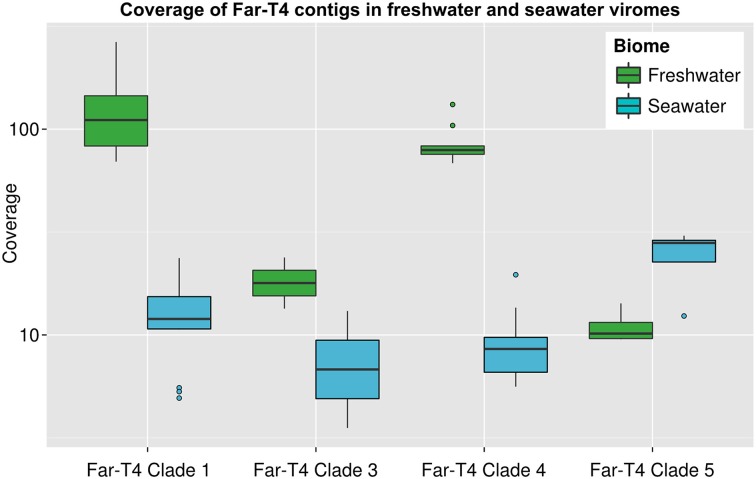
**Coverage of different Far-T4 clades in seawater and freshwater viral metagenomes**. For each category of virome (freshwater and seawater), the coverage of each contig (i.e., number of bp similar to a contig normalized by the contig length) was computed from 2.5 millions of randomly selected reads from each virome. Coverage could not be assessed for Far-T4 clade 2 since no contigs are available for this clade.

Refining these analyses to require non-redundant recruitment to the newly available Far-T4 phage contigs suggested that clades one and four were more prevalent in freshwater viromes (*t*-test *p*-values < 10^−07^), and clades three and five not significantly differently covered between freshwater and seawater (Figure 4). The nucleotide identity of the recruited reads was on average 70% for all clades, with up to 100% matches from some freshwater viromes (Figure S7). As previously interpreted (e.g., Holmfeldt et al., 2013) and given what is known about wild T4 cyanophage populations (>95% Average Nucleotide Identity within a population, Deng et al., 2014), this suggests that phages related to Far-T4 are likely occurring in seawater, but the contigs assembled from Lake Pavin represent freshwater-specific populations.

### Assessing putative host(s) for some Far-T4 phages

Without a close isolated reference, assessing the putative host(s) of a virus only detected in metagenomes is often difficult. Currently, the most straight-forward *in silico* approach is to look for sequences similar to the newly described virus in microbial genomic datasets, either as prophage (viral genome integrated in the host's genome), as separate contig in a single-amplified genome from an infected cell, or as a CRISPR spacer (i.e., 15–50 bp viral sequence(s) stored in a microbial genome from past infections). Here, no sequence closely related to Far-T4 phages could be detected in public databases of microbial genomes: the most similar genome sequences display only 50–60% amino acid identity (hits from genomic fragments in *Proteobacteria* SAGs, NCBI gis 655454702 and 655453257), and best hits to CRISPR spacers still displayed 2 mismatches when only 100% matches can be trusted on such short nucleotide sequences (hits to a *Clostridia*: *Peptoclostridium difficile*, gi 484228666, and a *Bacilli*: *Streptococcus pneumoniae*, gi 452723578). Thus, no putative host could be predicted for Far-T4 phages based on a search of microbial genomic datasets.

Another approach available is to use genome composition similarities between viral genome(s) and reference microbial genomes (especially tetranucleotide frequencies, Pride et al., 2006). In the Far-T4 case, 3 sequences from the Far-T4 clade 1 display tetranucleotide frequencies close to the small *Alphaproteobacteria Ehrlichia chaffeensis* (Table 1), an obligate intracellular parasite mostly found in animals and ticks. Based on a previous evaluation on more than 15,000 known virus-host pairs, such similarity in tetranucleotide frequencies correspond to host family predictions 88 to 98% accurate, and in that case would link Far-T4 phages with small *Alphaproteobacteria* from the *Anaplasmataceae* family.

## Discussion

Because so few environmental microbes can be cultivated in the lab (Rappé and Giovannoni, 2003), most viruses infecting these microbes are still to be characterized. In the absence of isolated host, groups such as the Far-T4 phages, although seemingly abundant, could until recently only be studied through single marker genes analyses, either from short-read metagenomes (Roux et al., 2012) or PCR amplification (Comeau and Krisch, 2008). Here, HiSeq Illumina sequencing provided large genome fragments to expand our genomic context and understanding of this Far-T4 group. Specifically, these data help to (i) validate the existence and distribution of several Far-T4 clades based on multiple genes, (ii) evaluate genome organization in this group, and (iii) witness patterns of evolution on conserved genes.

The Far-T4 group, initially identified from marker genes, appears here to represent a set of phages that display T4 phage characteristics, but are distant from the known T4 phages by every marker evaluated. A comparison between Far-T4 contigs further validates the clade delineation based on single marker genes, and suggests that Gp23 is a very good marker for this extended T4 family. Genome comparison also revealed an important genetic diversity within this group: even if all Far-T4 contigs form a monophyletic clade, they only share a few “core” genes. The consistency in clade delineation using phylogenetic markers and gene content analysis associated with the overall conserved modular genome structure indicates that all Far-T4 likely derived from a (likely distant) common ancestor, and most of the “variable” genes (genes outside of the highly conserved core genes) have either diverged too much to recover any similarity or were subject to genome re-arrangement and/or horizontal gene transfer.

From the genome context perspective, these newly available Far-T4 phage genome fragments clearly evidence that *Rhodothermus marinus* phage RM378, the closest cultured representative of the Far-T4 phages, seems anomalously representative of the Far-T4 at best. Specifically, the Far-T4 phages have similar genomic organizational properties to the Near- and Cyano-T4 phages, while phage RM378 does not. Far-T4 phage genome fragments also display a handful of the T4 “core” genes (10 out of 38), including all major virion-related proteins (notably the major capsid, portal, terminase, and tail proteins) and main replication proteins (DNA polymerase, primase/helicase, and both subunits of ribonucleotide reductase). This suggests that Far-T4 phages will harbor T4-like morphology (as does RM378) and probably T4-like replication mechanism. However, the absence of detection of T4 “core” genes linked to virus-host interactions and transcription regulation indicates that Far-T4 host interaction dynamics, transcription patterns, and infection cycle might differ from the T4 phages, and that their characterization will require further experiments beyond sequence analysis. Alternatively, some T4 “core” genes might also be missing because the Far-T4 contigs represent only partial genomes.

Finally, metagenomic fragment recruitment analyses on these new genomic fragments establish these Far-T4 phages as being widely distributed around the world in aquatic systems—freshwater and seawater. Interestingly, Far-T4 phages were thought to be absent from freshwater systems due to the lack of PCR-based amplicons using newly optimized primer sets (Comeau and Krisch, 2008), however this directly reflects the fact that the PCR primers were designed from seawater sequences, and are not able to amplify Far-T4 sequences assembled from freshwater. This difficulty in designing universal primers, even for specific target groups, is relatively well known in microbial ecology and a source of controversy and debate in trying to establish quantitative viral ecology (Sullivan et al., 2008), and viromics may better represent viral abundances, at least for dsDNA phages (Duhaime and Sullivan, 2012; Sullivan, 2015).

Stepping back, this and related studies that leverage viromics to characterize new viruses (e.g., Rosario et al., 2009a; Emerson et al., 2012; Dutilh et al., 2014) help illustrate the inferential advances and remaining challenges of the approach. Specifically, viromics clearly enables assembly of new phage groups that have eluded cultivation so far, as well as fragment recruitment analytical capabilities to provide environmental context for newly available reference genomes. Yet two main challenges remained: (i) assembling complete and accurate genomes from complex communities, and (ii) extracting information beyond this genome sequence, especially the host(s), quantification in different ecosystems, and characterization of infection cycle of the newly described virus, all required to really assess the potential impact of a virus on ecosystems.

Rigorously evaluating the quality of metagenomic assemblies (i.e., do contigs represent real consensus genomes or *in silico* generated chimeras) remains fundamentally problematic especially since no gold standard metrics (e.g., what is real?) are readily apparent with newly discovered environmental data (Charuvaka and Rangwala, 2011; Luo et al., 2012; Vázquez-Castellanos et al., 2014). For the most abundant viruses, the high coverage provided by most recent sequencing technologies seems to lead to accurate and reproducible assemblies, as testified by PCR confirmation of metagenome-based assemblies (Dutilh et al., 2014) or the recovery of identical contigs from separate samples in this study. However, such high coverage is not yet available for members of the “rare virosphere.” Several workarounds have been proposed to access this rare virosphere such as single cell viromics (Allen et al., 2011), viral tagging (Deng et al., 2014) or targeted viromics (Brum et al., 2013). Additionally, even if robust assemblies of large genome fragments are now possible, the assembly of complete genomes from complex communities is still relatively rare, notably because of the presence of repeat regions and highly conserved sequences in phage genomes, which generate ambiguous cases that assemblers can not resolve with short reads alone. Several approaches can help to close the genomes such as PCR amplification based on the partially assembled genomes (Culley et al., 2007), or the use of a mix of long and short reads from the same sample as already done for microbial genomes (Boisvert, 2010).

The second major challenge of virus discovery through metagenomics is the extrapolation of characteristics beyond the genome sequence, with the most important one being the host range of the new virus. Except for the cases where a sequence identical (or nearly-identical) to the new virus is available in a sequenced microbial genome, assessing putative host is a tricky process. In the Far-T4 example, the putative host predicted by the different methods are all spurious, and non consistent. An *in silico* identification of putative host groups through genome composition (here tetranucleotide frequency) seems to be promising (Roux et al., in revision), and should be more and more efficient with the increasing coverage of microbial genome sequence space, as well as in cases where both a microbial and a viral metagenome are available from the same sample. However, such methodology will only provide a prediction of putative host that has to be verified by complimentary experiments like phageFISH (Allers et al., 2013), microfluidic digital PCR (Tadmor et al., 2011), or viral tagging (Deng et al., 2014) (reviewed in Dang and Sullivan, 2014; Brum and Sullivan, 2015). Assessing the impact of a virus also requires its quantification in different types of samples. If such quantification is now available for dsDNA viruses through linker-amplified metagenomes, there are no quantitative methodologies yet available for ssDNA and RNA viromes (Duhaime and Sullivan, 2012; Brum and Sullivan, 2015). Finally, the characterization of infection cycle through sequence analysis alone is hampered by the high number of “novelty” in each new viral genome (resulting in a lot of “hypothetical genes”,) even for phages that are related to well-characterized isolates as the Far-T4 are from the T4 phages. Eventually, using these newly described genome sequences as anchors or probes for *in-situ* approaches like phageFISH (Allers et al., 2013), meta-transcriptomics and viral meta-proteomics will be decisive to advance our knowledge of viral diversity beyond a first description of their genome and really characterize these new viruses.

## Materials and methods

### Virome generation and assembly

The procedure used to generate viromes is the same as previously described (Roux et al., 2012). Briefly, each water sample was filtered on 0.22 μm, and virus-like particles were concentrated by tangential ultrafiltration and PEG precipitation (Colombet et al., 2007). These concentrates were treated with DNaseI to remove external fragments, before encapsidated DNA was freed via a thermal shock, purified with a QIAamp DNA mini kit (Qiagen), and randomly amplified with the phi29 polymerase using random hexamer primers (Genomiphi Kit, GE Healthcare). In a first study of two lakes (Lake Pavin and Lake Bourget), twenty liters of water were sampled at a 5 m depth in June and July 2008, and subjected (after preparation steps) to a single pyrosequencing run by GATC Biotech (Germany) using a 454 Life Sciences Genome Sequencer GS-FLX (Roux et al., 2012). Both virome read sets are available on the Short Read Archive (accession number: ERP000339). For this study, two samples were taken at 4 and 8 m on Lake Pavin in July 2013 and sequenced (after preparation) with Illumina HiSeq (GATC Biotech, Germany).

For 454 viromes, reads were first clustered using Uclust (Edgar, 2010) at a 100% identity level, in order to remove duplicate sequences, and sequence assembly was conducted with Newbler using threshold of 90% identity on at least 35 nucleotides. Illlumina sequences were trimmed by quality score (cutoff at 30 using FASTX v0.0.13) and then assembled using IDBA-UD (Peng et al., 2012).

### Far-T4 contig selection

The major capsid protein Gp23, present in all T4 phages, is the only marker available for the Far-T4 group (Comeau and Krisch, 2008). First, all T4 sequences were identified by screening contigs for the presence of Gp23. Second, a phylogenetic tree of all virome sequences similar to Gp23 was computed in order to distinguish Far-T4 from other T4 phages and from putative false positive sequences. Thus, only Gp23 sequences found near the known Far-T4 sequences on the tree and displaying both N-terminal domain (coordinates 122–162) and C-terminal domain (coordinates 735–766) were kept (see Figure S5 for a complete view of the multiple alignment). For Illumina viromes, only genes detected on the contigs longer than 10 kb were selected to limit the total number of sequences in the analysis. All identified Far-T4 contigs were then automatically annotated with Metavir 2 (Roux et al., 2014b), which includes a gene prediction with Metagene Annotator (Noguchi et al., 2006), blastp comparison to NCBI Refseq genomes, and HMMER comparison with PFAM profiles (Punta et al., 2012), and are publicly available on Metavir (http://metavir-meb.univ-bpclermont.fr/) under project “FarT4 / Far-T4 Lake Pavin”.

### Sequence analysis

Using proteins predicted from the Far-T4 contigs identified, phylogenies were inferred for different T4 phages conserved genes, namely the ones coding for the major capsid protein Gp23 (Figure 1), the portal protein Gp20, the large subunit of the terminase Gp17 and PhoH (Figure S1). For all these trees, reference sequences were obtained from the NCBI Refseq database of complete phage genomes, except for the g23 PCR amplicons that were obtained from the NCBI Genbank database. All phylogenies were based on (predicted) protein sequences.

Mutiple alignments were computed with Muscle (Edgar, 2004) and manually curated. The Gp23 multiple alignment was trimmed around the PCR amplicon boundaries to avoid artificially increased distances between sequences. FastTree2 (Price et al., 2010) was used to generate maximum-likelihood trees (WAG model). For all trees, all branches with bootstrap score lower than 50 were collapsed. The tree figures were edited with Itol (Letunic and Bork, 2007). The position of the root between the Far-T4 and all the other T4-like phages was determined by including an outgroup including *Spounavirinae* (another subfamily of *Myoviridae*).

### Genome fragment comparison

To evaluate the “novelty” of Far-T4 contigs for each clade, the proportion of genes affiliated to NR, only similar to another Far-T4 contig or unique to a contig were calculated (Figure S2). A clustering of contigs was based on a blastp comparison of all vs. all predicted proteins from contigs. Genes were considered as shared when they displayed a blastp hit with a bit score greater than 50 and an *e*-value lower than 0.001. A proportion of shared genes between pairs of contigs was then computed as the number of genes shared between the two contigs divided by the length of the shortest contig. The cluster heatmap was computed in R with pheatmap package. For this analysis, duplicate contigs (*i.e.*, contigs 100% identical assembled from different samples) were excluded.

Finally, for the 12 contigs longer than 25 kb, the sequence comparison and map generation was performed using blastn (bit score >50) and Easyfig version 2.1 (Sullivan et al., 2011).

### Major capsid protein alignment and structure analysis

Jalview (Waterhouse et al., 2009) was used to display the multiple alignment of Gp23 as well as calculating residue conservation and consensus sequence. PaML (Yang, 2007) was used to calculate the dN/dS ratios and their associated likelihood value. Statistical tests were computed to detect the significance of likelihoods differences between evolutionary hypothesis as in (Zhang et al., 2005): (i) a single dN/dS ratio for all positions and all sequences, (ii) two dN/dS categories for all sequences, one linked to conserved sites, and one with sites under relaxed selection pressure, (iii) three different dN/dS for all sequences, one for conserved sites, one for relaxed selection sites, and one for sites under positive selections, and (iv) two different dN/dS for all sites, one for branches in the Far-T4 subtree, the other for all other branches (Table S1).

Secondary structures were predicted with I-Tasser (Roy et al., 2010) from the Gp23 sequence of contig Pavin_2013_4m-8. I-Tasser was also used to generate 3D models for representative sequences of each clade (based on the primary sequence contigs Pavin_2013_4m-8, Pavin_2013_4m-10, Pavin_2013_4m_77 and Pavin_2013_4m_62), based on the known structure of the conserved domain of the T4 vertex protein, which is shared with the T4 major capsid protein. For each major capsid protein, the stereochemical quality of each of the five models generated by I-Tasser for each sequence was assessed with ProSA-web (Wiederstein and Sippl, 2007), and the model with the best quality score on ProSA was kept. Model quality ranged from −5.4 to −7.56, in the range of X-Ray confirmed models for proteins of similar sizes (Figure S6). UCSF Chimera was used to display the different models as well as sequence conservation information (Pettersen et al., 2004).

### Detection of Far-T4 phages in other viral metagenomes

Sequences similar to the large Far-T4 contigs assembled from Lake Pavin Illumina viromes, were searched in a large collection of viromes using tblastx (bit score >50, *e*-value < 0.001). Sequences similar to Far-T4 contigs were detected in seawater viromes from the Pacific Ocean Viromes (POV, Hurwitz and Sullivan, 2013), the Indian Ocean (Williamson et al., 2012), and the Atlantic Ocean (Chesapeake Bay, part of the GOS dataset Yooseph et al., 2007). Far-T4 sequences were also detected in several freshwater viromes from lakes in Europe (Roux et al., 2012), in Asia (Ge et al., 2013; Tseng et al., 2013), Antarctica (López-bueno et al., 2009), and in freshwater ponds in the USA (Rosario et al., 2009b). Conversely, no sequences similar to the Far-T4 were detected in other types of samples including human gut (Kim et al., 2011; Minot et al., 2012a), airborne samples (Whon et al., 2012) or plant samples (Coetzee et al., 2010).

Recruitment plots were generated with ggplot2 module in R, considering only BLAST hits with an amino-acid identity higher than 60% (in addition to bit score > 50 and *e*-value < 0.001). Coverage was calculated as the log10 of the number of reads mapped to the contig on sliding windows corresponding to a 30th of the contig length (i.e., for a contig of 30 kb, sliding windows of 1 kb would be used).

### Host prediction

Prophages or phages infecting single-cells (SAGs) closely related to Far-T4 were searched in microbial draft genomes by comparing predicted proteins from Far-T4 to the bacterial and archaeal genomes in Refseq and WGS NCBI database (blastp, bit score >50 and *e*-value < 0.001). In addition, CRISPR spacers were predicted on the bacterial and archaeal genomes available at Refseq and WGS (as of January 2014) with CRT (Bland et al., 2007) and then compared to the Far-T4 contigs with blastn. As CRISPR spacers are short sequences, more stringent thresholds were applied: only hits that covered more than 80% of the CRISPR spacer with more than 90% of nucleotide identity were considered significant. Three matches were identified: contigs Pavin_2013_4m_335 and Pavin_2013_4m_328 were similar to a CRISPR spacer from a *Streptococcus pneumoniae* genome (gi 452723578) at 92% of identity, and contig Pavin_2013_4m_8 matches a CRISPR spacer from another *Firmicutes*, *Peptoclostridium difficile* (gi 484228666), at 90% of identity.

Host prediction based on genomic signature was also computed using tetranucleotide frequency as in (Roux et al., in revision). First, tetranucleotide frequency vectors were calculated for each Far-T4 contig with Jellyfish (Marçais and Kingsford, 2011). The euclidean distance between these vectors and the tetranucleotide frequency vectors from bacterial and archaeal genomes in Refseq and WGS (as of January 2014) were then calculated. A previous analysis of more than 12,000 virus-host pairs indicated that in the absence of the exact host species in the database (which is the most likely case for freshwater viruses), host family could be predicted with 95% of success if the distance between virus and host tetranucleotide frequency vectors was below 4.10^−04^, and with 84% of success if it was between 4.10^−04^ and 1.10^−03^. For the Far-T4 phages, we could not detect any correspondence between Far-T4 contigs and microbial genomes displaying a distance lower than 2.10^−04^, but three contigs from Clade 1 displayed a distance of 4,7.10^−04^ with genomes of *Ehrlichia chaffeensis* (two matching str. Arkansas–NC_007799.1, and one matching str. Sapulpa–GCF_000167655.1).

### Conflict of interest statement

The authors declare that the research was conducted in the absence of any commercial or financial relationships that could be construed as a potential conflict of interest.
