# Supplementary material for: Genomic characteristics and environmental distributions of the uncultivated Far-T4 phages
Source: Front Microbiol. 2015 Mar 16;6:199. doi: 10.3389/fmicb.2015.00199 (PMC4360716; doi:10.3389/fmicb.2015.00199)
Supplement: Supplementary file 1 [file Presentation1.PDF]

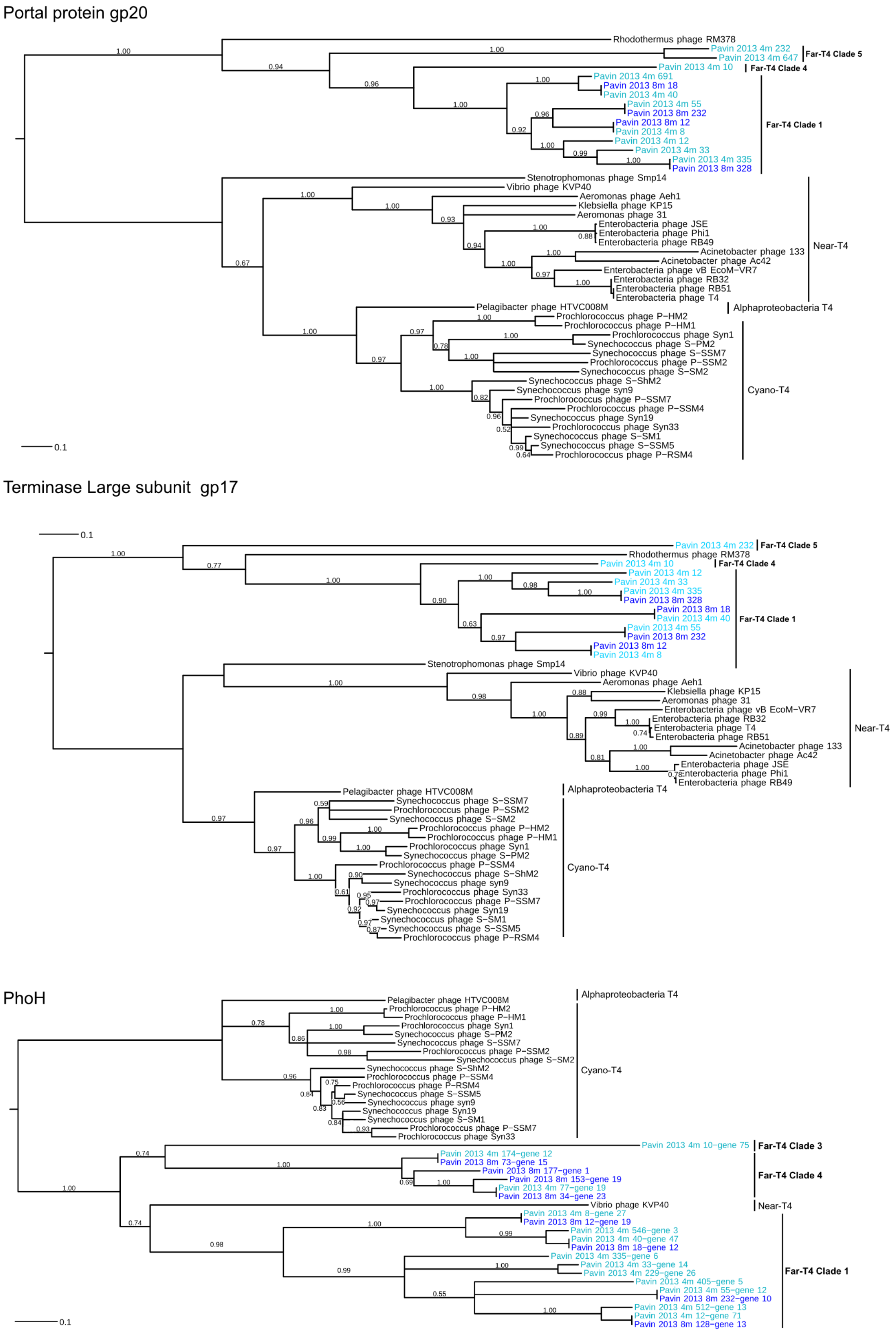

Fig S1. **Phylogenetic trees computed from markers Gp20 and Gp17, and putative marker PhoH.** For each tree, nodes with bootstrap support lower than 50 were collapsed. Sequences from Lake Pavin are in light and dark blue for 4m and 8m samples respectively. No other dataset presented these marker genes on the same contig as a Far-T4 affiliated Gp23.

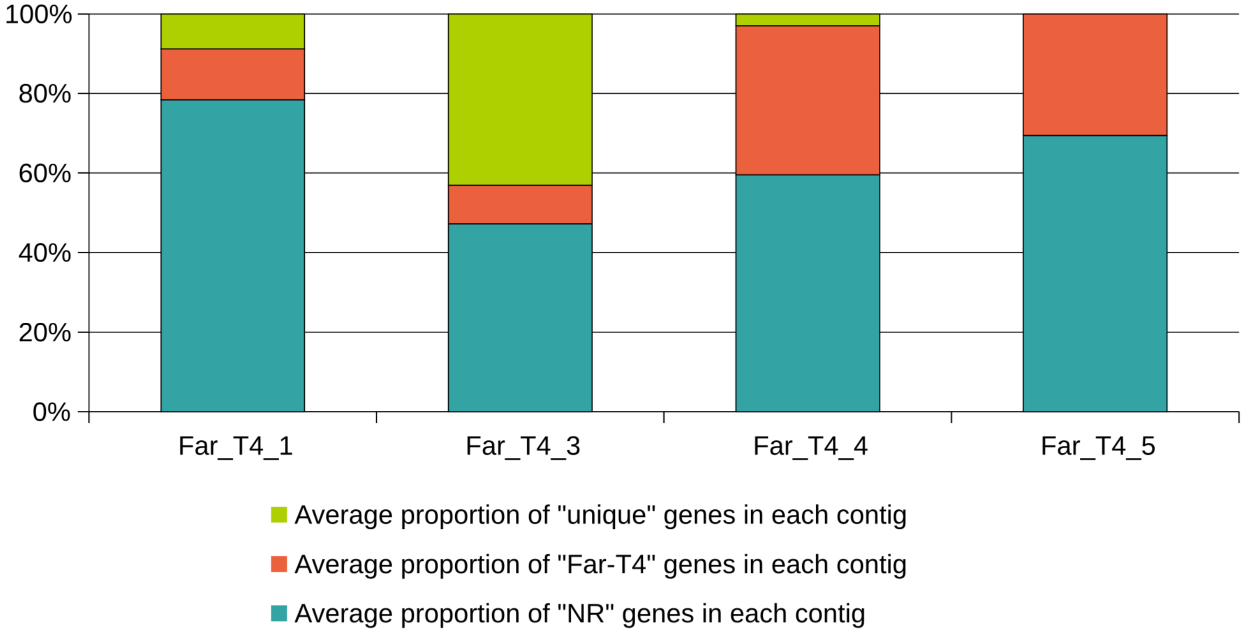

Fig S2. **Affiliation of genes from the different Far-T4 clades.** Genes can be either affiliated to “NR” if they display significant similarity to a sequence from the nr database, “Far-T4 only” is the only significant similarity detected is with another Far-T4 contig, or “Unique” if no significant similarity was detected. These proportions were calculated for each contig longer than 25kb with duplicated contigs (*i.e.* contigs 100% identical assembled from different samples) excluded.

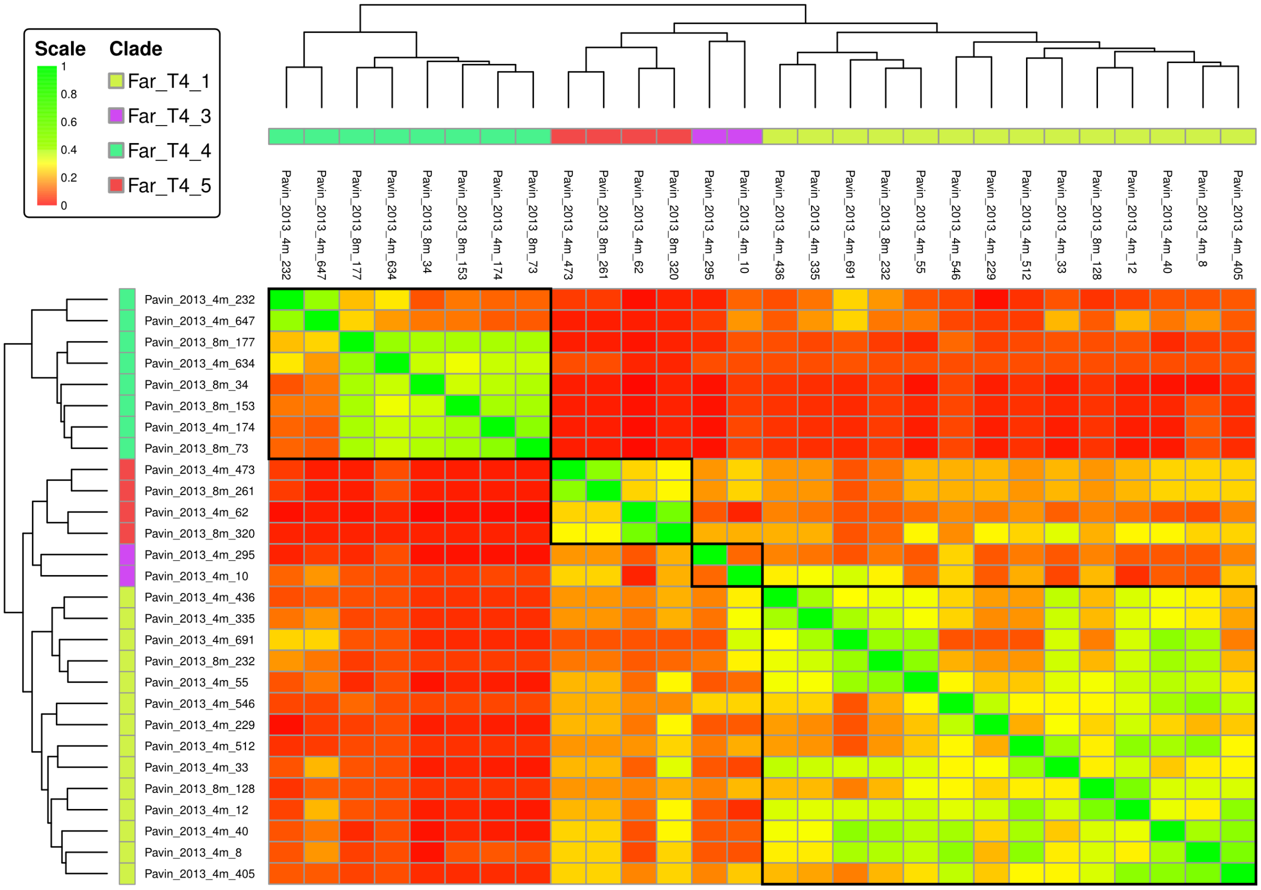

Fig S3. **Heatmap clustering Far-T4 contigs based on a proportion of shared genes.** The proportion of shared genes between a pair of contigs was computed as the number of similar genes detected between the two contigs divided by the number of genes of the shortest contig.

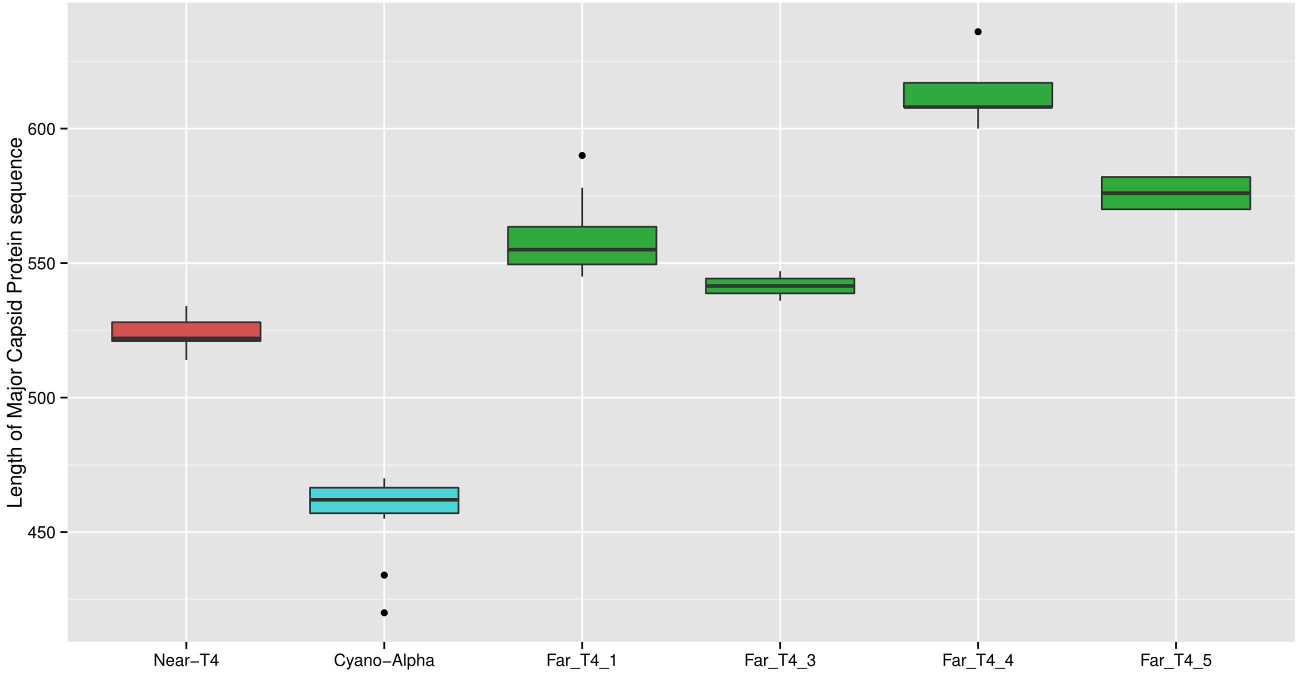

Fig S4. **Distribution of length of Gp23 predicted proteins for each T4 clade.** Upper and lower "hinges" correspond to the first and third quartiles (the 25th and 75th percentiles), while outliers are displayed as points (values beyond 1.5 * Inter-Quartile Range of the hinge).

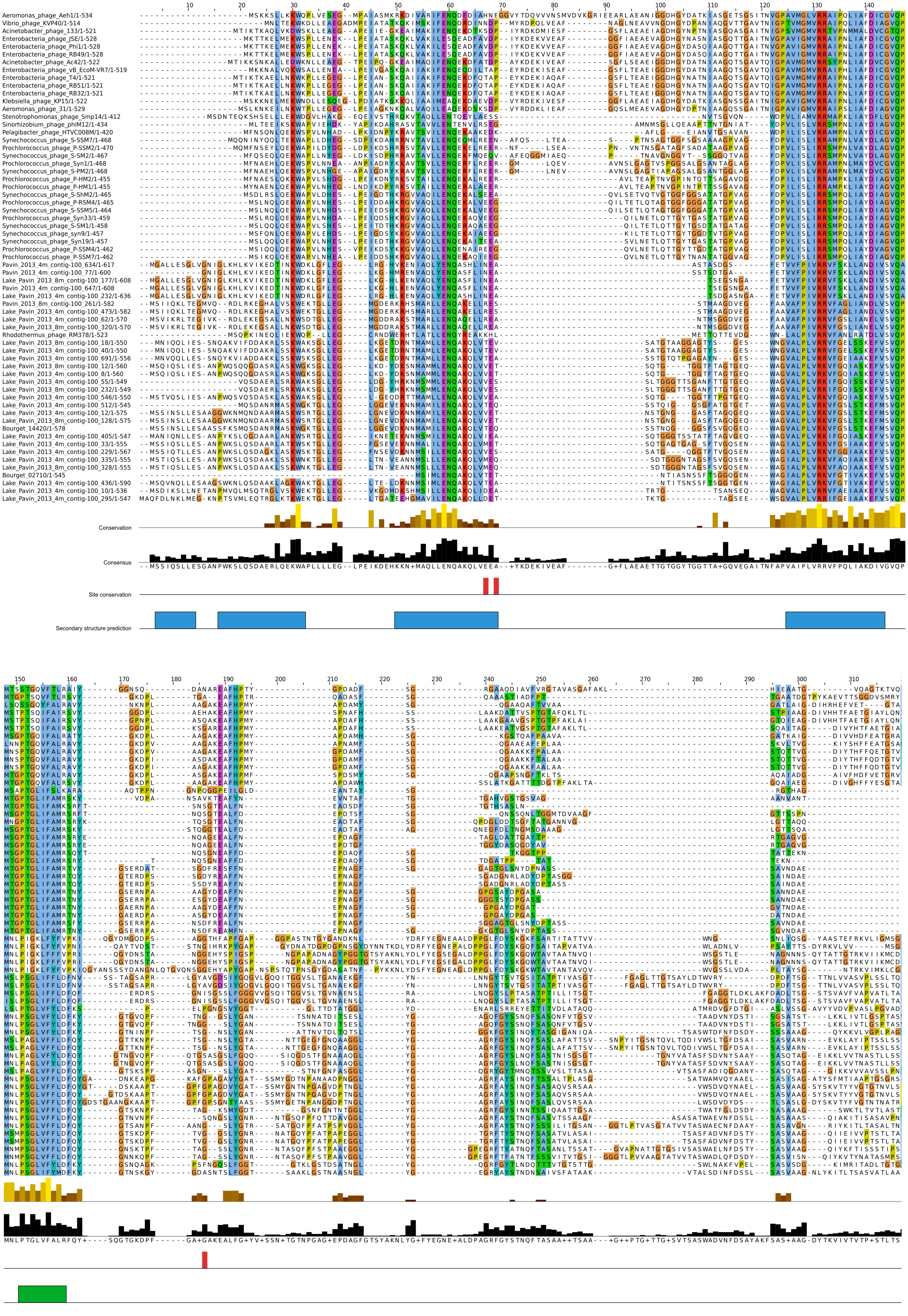
Aeromonas_phage_Aeh1/1-534
Vibrio_phage_KVP40/1-514
Acinetobacter_phage_133/1-521
Enterobacteria_phage_JSE/1-528
Enterobacteria_phage_Phi1/1-528
Enterobacteria_phage_RB49/1-528
Acinetobacter_phage_Ac42/1-522
Enterobacteria_phage_vB_EcoM-VR7/1-519
Enterobacteria_phage_T4/1-521
Enterobacteria_phage_RB51/1-521
Enterobacteria_phage_RB32/1-521
Klebsiella_phage_KP15/1-522
Aeromonas_phage_31/1-529
Stenotrophomonas_phage_Smp14/1-412
Sinorhizobium_phage_phiM12/1-434
Pelagibacter_phage_HTVC008M/1-420
Synechococcus_phage_S-SSM7/1-468
Prochlorococcus_phage_P-SSM2/1-470
Synechococcus_phage_S-SM2/1-467
Prochlorococcus_phage_Syn1/1-468
Synechococcus_phage_S-PM2/1-468
Prochlorococcus_phage_P-HM2/1-455
Prochlorococcus_phage_P-HM1/1-455
Synechococcus_phage_S-ShM2/1-465
Prochlorococcus_phage_P-RSM4/1-465
Synechococcus_phage_S-SSM5/1-464
Prochlorococcus_phage_Syn33/1-459
Synechococcus_phage_S-SM1/1-458
Synechococcus_phage_syn9/1-457
Synechococcus_phage_Syn19/1-457
Prochlorococcus_phage_P-SSM4/1-462
Prochlorococcus_phage_P-SSM7/1-462
Pavin_2013_4m_contig-100_634/1-617
Pavin_2013_4m_contig-100_77/1-600
Lake_Pavin_2013_8m_contig-100_177/1-608
Pavin_2013_4m_contig-100_647/1-608
Lake_Pavin_2013_4m_contig-100_232/1-636
Pavin_2013_8m_contig-100_261/1-582
Lake_Pavin_2013_4m_contig-100_473/1-582
Lake_Pavin_2013_4m_contig-100_62/1-570
Lake_Pavin_2013_8m_contig-100_320/1-570
Rhodothermus_phage_RM378/1-523
Lake_Pavin_2013_8m_contig-100_18/1-550
Lake_Pavin_2013_4m_contig-100_40/1-550
Lake_Pavin_2013_4m_contig-100_691/1-556
Lake_Pavin_2013_8m_contig-100_12/1-560
Lake_Pavin_2013_4m_contig-100_8/1-560
Lake_Pavin_2013_4m_contig-100_55/1-549
Lake_Pavin_2013_8m_contig-100_232/1-549
Lake_Pavin_2013_4m_contig-100_546/1-550
Lake_Pavin_2013_4m_contig-100_512/1-545
Lake_Pavin_2013_4m_contig-100_12/1-575
Lake_Pavin_2013_8m_contig-100_128/1-575
Bourget_14420/1-578
Lake_Pavin_2013_4m_contig-100_405/1-547
Lake_Pavin_2013_4m_contig-100_33/1-555
Lake_Pavin_2013_4m_contig-100_229/1-567
Lake_Pavin_2013_4m_contig-100_335/1-555
Lake_Pavin_2013_4m_contig-100_328/1-555
Bourget_02710/1-545
Lake_Pavin_2013_4m_contig-100_436/1-590
Lake_Pavin_2013_4m_contig-100_10/1-536
Lake_Pavin_2013_4m_contig-100_295/1-547
Conservation
Consensus
Site conservation
Secondary structure prediction

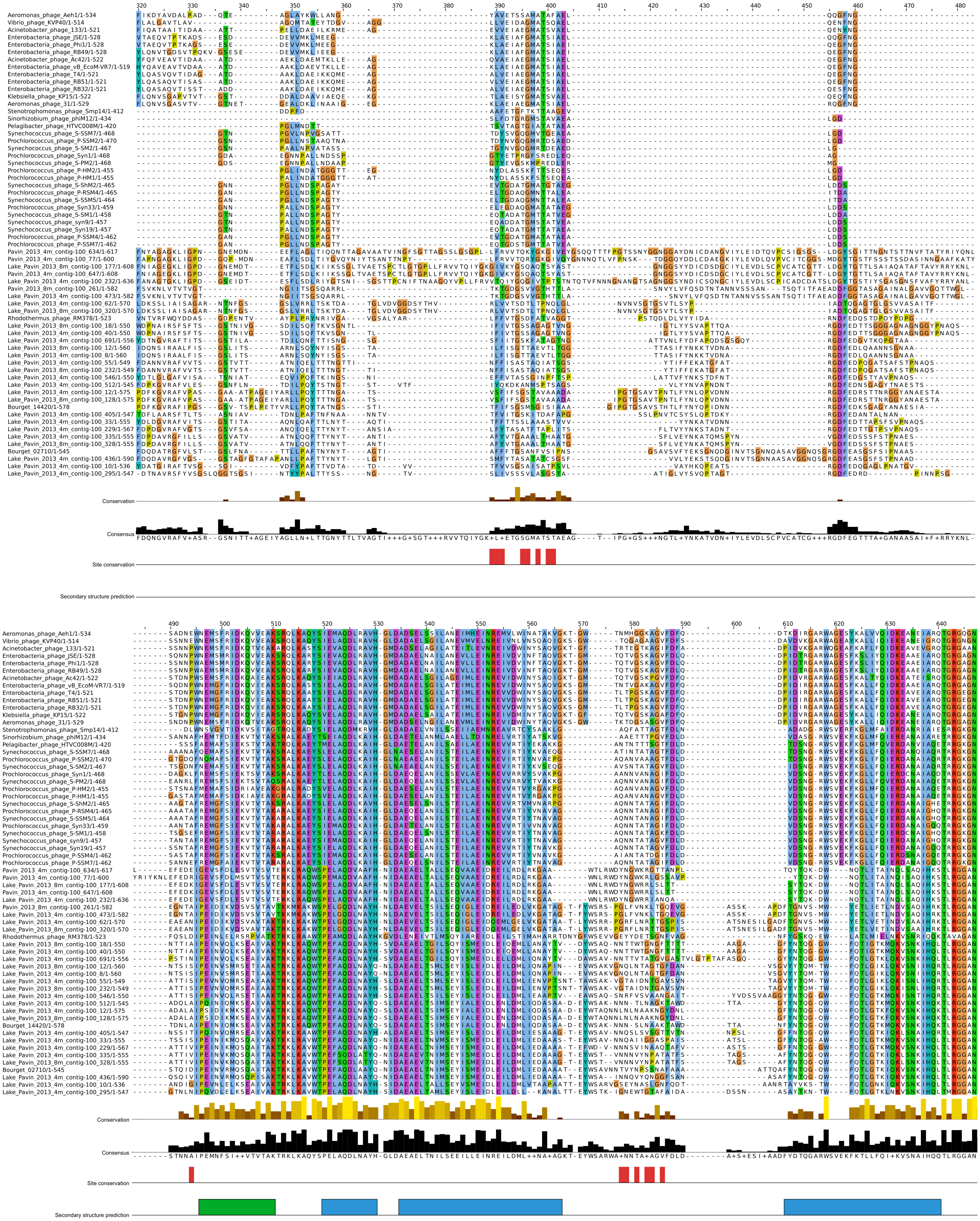

Conservation
Consensus
Site conservation
Secondary structure prediction

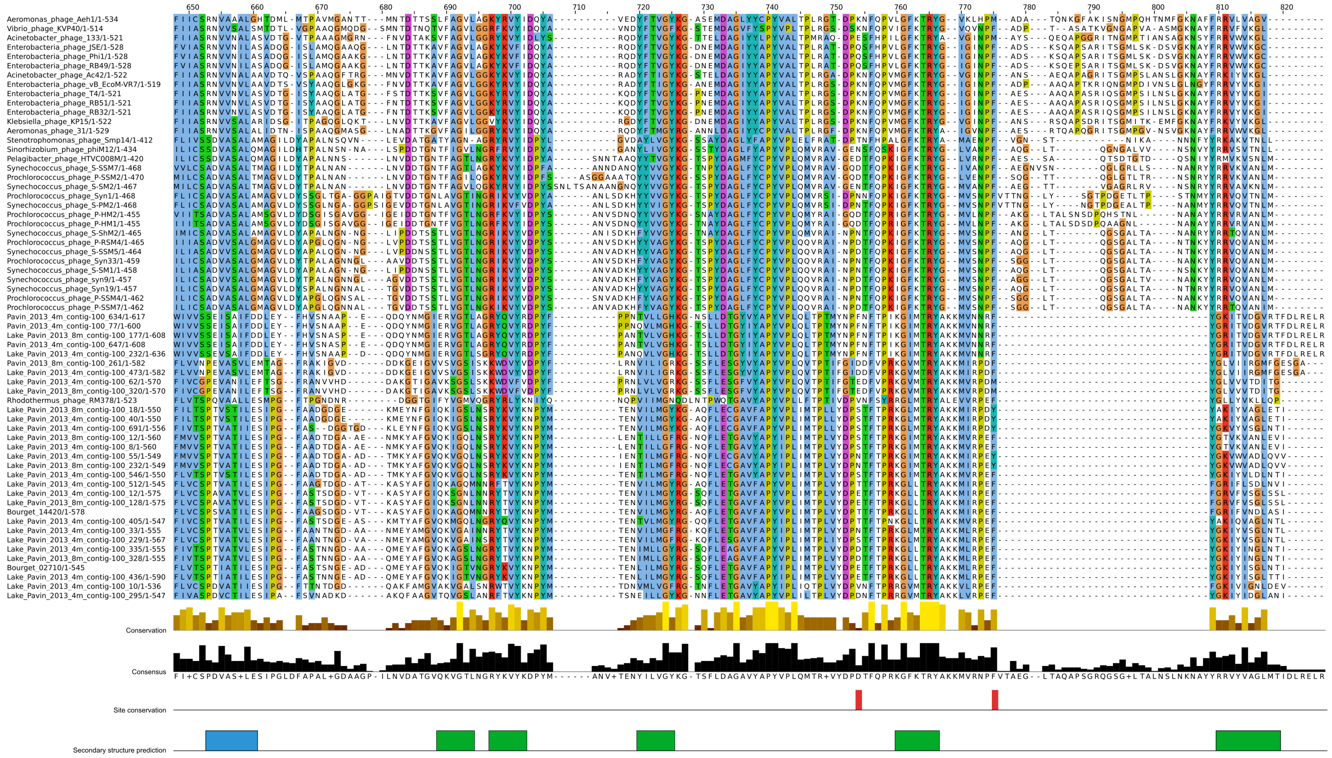

Fig S5. **Multiple alignment of Gp23 protein sequences.** The level of sequence conservation and consensus sequence as calculated by Jalview, the category of site in the dN/dS analysis (sites under statistically lowered selection pressure are indicated in red, only sites without any gap were included in this analysis), and the secondary structure prediction from I-Tasser (blue: alpha helix, green: beta strands), are indicated below each position.

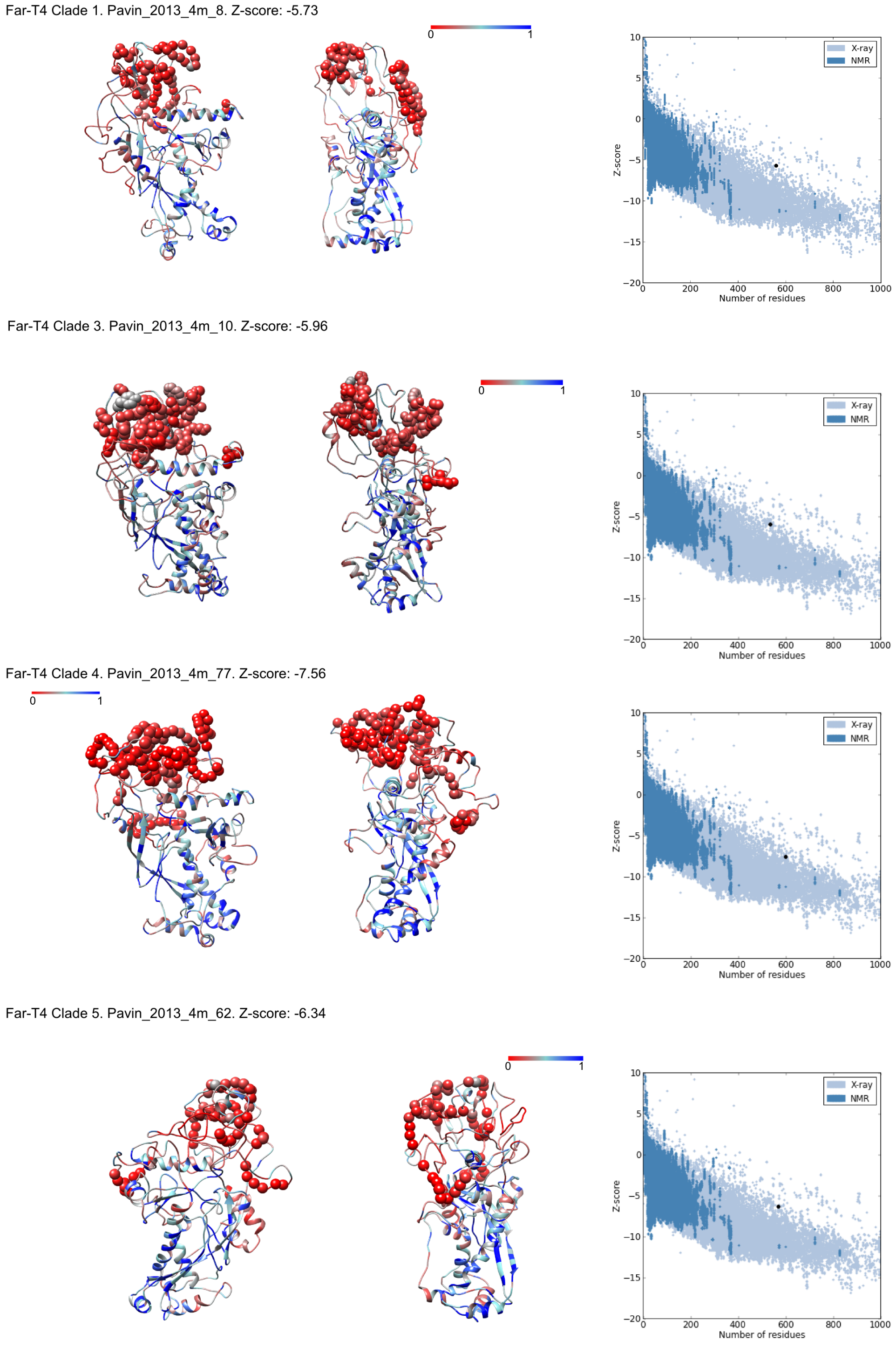

Fig S6. **Predicted structures and associated model quality for the major capsid protein of different Far-T4 clades.** One representative was selected for structure prediction of its major capsid protein for each Far-T4 clade. For each of these sequences, the best-evaluated (lower Z-score in ProSAweb) predicted structure is displayed (two views rotated by 90° through the y-axis) colored according to the residue conservation, alongside the model evaluation result by ProSAweb.

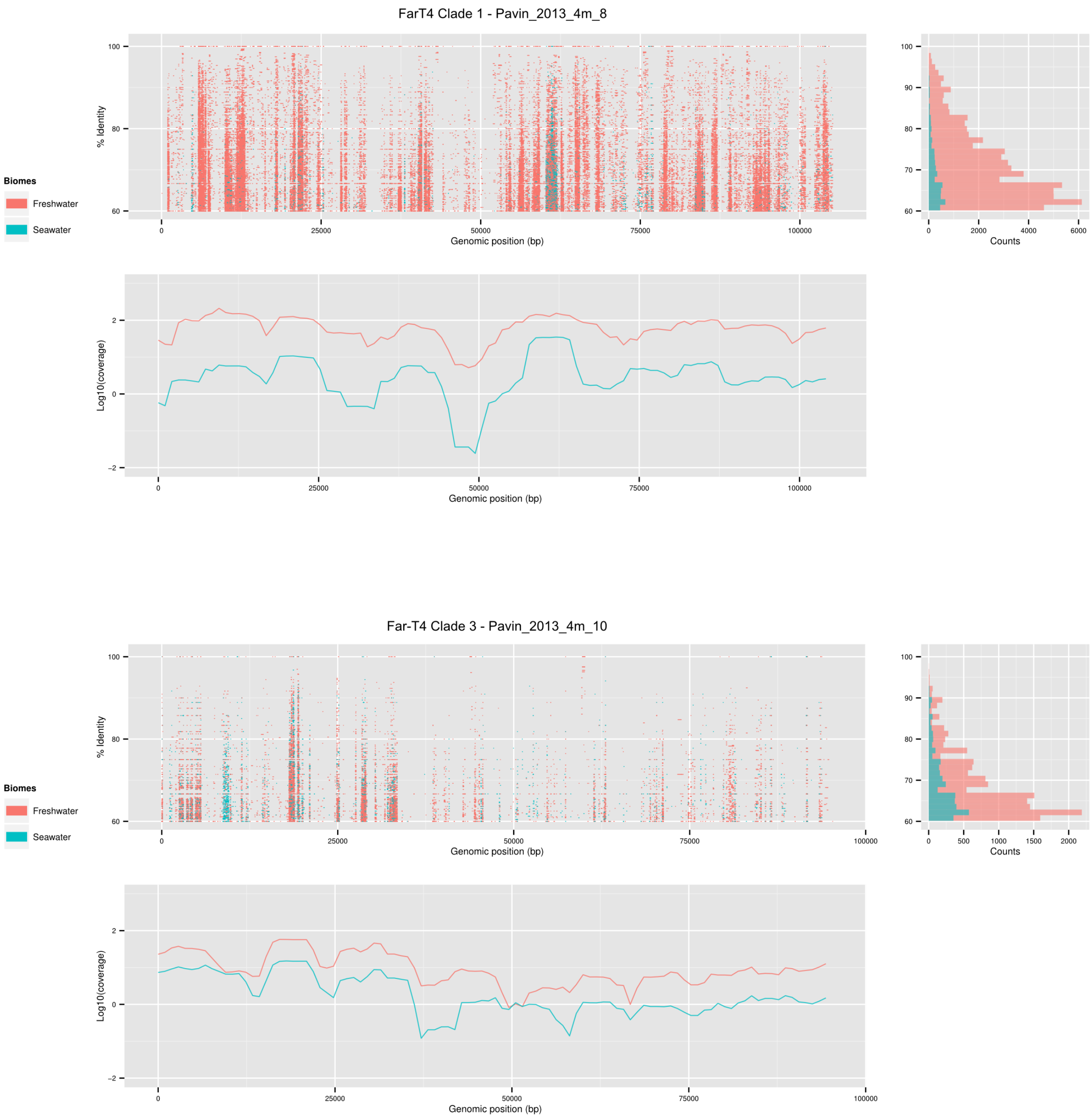

Fig S7. **Recruitment plots of freshwater and seawater virome reads on Far-T4 contigs.** The longest contig was chosen as the representative of each Far-T4 clade. For each sequence, a set of three plots displays the invidiual recruitment of reads (on top), the corresponding log-coverage (bottom) and corresponding distribution of reads according to their identity level to the contig (right). Coverage values are calculated with a sliding window size set at 1/30th of the contig length.

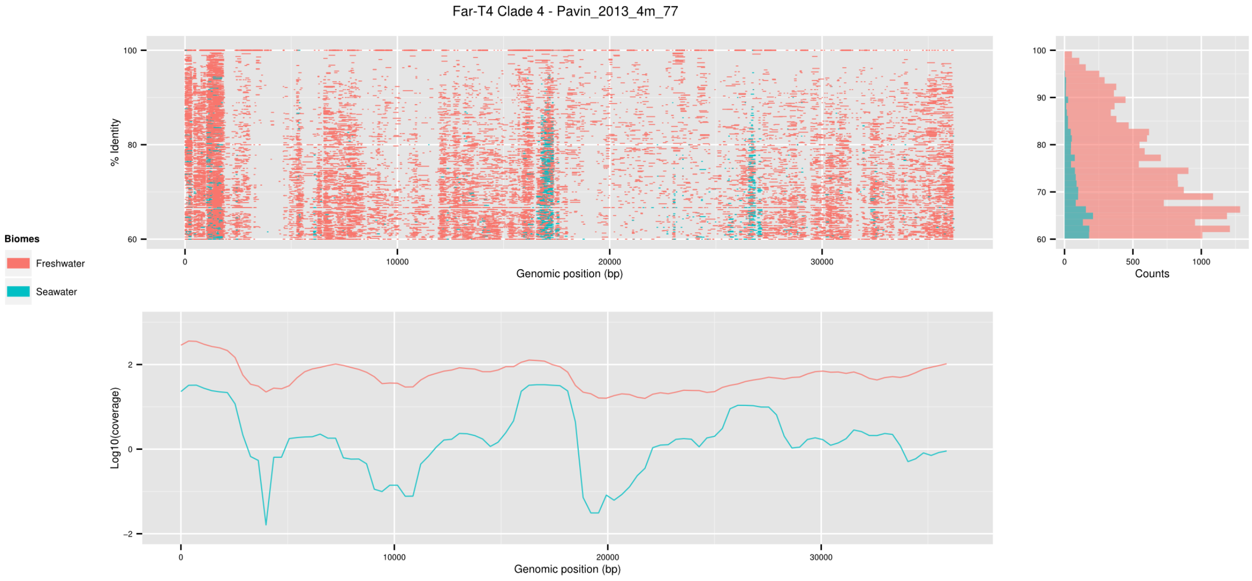
Far-T4 Clade 4 - Pavin_2013_4m_77
% Identity
Genomic position (bp)
Counts
Biomes
Freshwater
Seawater
Log10(coverage)
Genomic position (bp)

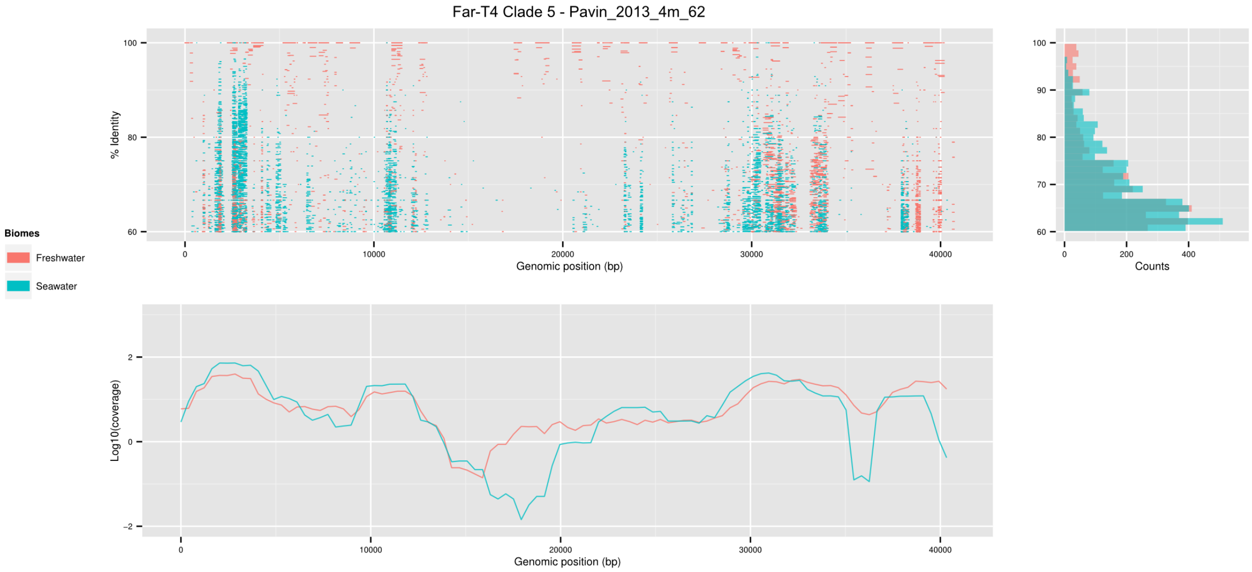
Far-T4 Clade 5 - Pavin_2013_4m_62
% Identity
Genomic position (bp)
Counts
Biomes
Freshwater
Seawater
Log10(coverage)
Genomic position (bp)

Model	Tree likelihood	ω1	ω2	ω3	Distance to H0 (p-value)	Distance to H1 (p-value)
H0: One category of site, same ω for all sites	-30 408.87	0.1037	-	-	-	-
H1: Two categories of site, with different values of ω (ω1 and ω2)	-30 092.59	0.09289	1	-	471.09 (1.86E-104)	-
H2: Three categories of site, with different values of ω (ω1 and ω2 and ω3)	-30 092.59	0.09289	1	1	471.09 (8.79E-102)	0 (1)
H3 : Two different values of ω, one (ω1) for the subtree including all Far-T4, the other (ω2) for the subtree including the other T4-like phages	-30 402.11	0.1181	0.09273	-	13.51 (2.37E-04)	-

Table S1. **Likelihood ratio test for gp23 multiple alignments.** Codeml was used to estimate dn/ds ratios (i.e. ω) from the multiple alignment of gp23 presented in figure S5. Four scenarios were tested: (i) only one value of ω for all positions in the alignment (model=0), (ii) two different categories of positions with one under neutral evolution (ω=1) and one under selection (model=1), (iii) three categories of sites under positive, negative or neutral evolution (model =2) and (iv) two different dn/ds for all sites, one for branches in the Far-T4 subtree, the other for all other branches (Table S1).

Virome Id	Sample	Total number of hits to Far-T4 phages	Number of hits to Far-T4 Clade 1	Number of hits to Far-T4 Clade 3	Number of hits to Far-T4 Clade 4	Number of hits to Far-T4 Clade 5
MET7	Lake Bourget (France)	37 811	29 872	2 348	3 934	790
POV-STCS	Scripps Pier (Pacific Ocean)	14 083	8 180	1 384	1 870	2 296
POV-SFDS	Scripps Pier (Pacific Ocean)	12 254	7 710	1 646	1 378	1 051
MET6	Lake Pavin (France)	11 387	6 562	1 024	2 812	612
POV-SFSS	Scripps Pier (Pacific Ocean)	10 505	6 765	1 339	941	950
POV-SFCS	Scripps Pier (Pacific Ocean)	10 042	6 427	1 079	1 092	1 051
GS117	Indian Ocean	7 895	4 311	690	1 462	1 213
FTR_Jan2009	Feitsui Reservoir (Taiwan)	5 962	2 245	908	52	93
GS112	Indian Ocean	5 377	2 885	306	1 271	809
POV-M1CS	MBARI transect (Pacific Ocean)	4 917	3 112	542	333	799
POV-M2MS	MBARI transect (Pacific Ocean)	4 653	2 902	484	485	666
GS108	Indian Ocean	4 232	1 969	342	867	962
POV-LJ12S	LineP Transect (Pacific Ocean)	4 145	2 664	507	204	193
East_Lake_Wuhan_China_Aug2009	East Lake (Wuhan, China)	3 633	2 039	908	382	153
FTR_Jul2007	Feitsui Reservoir (Taiwan)	3 545	2 912	158	300	69
POV-LA26S	LineP Transect (Pacific Ocean)	3 138	1 762	905	204	105
GS122	Indian Ocean	2 816	1 770	131	478	392
POV-LJ4A	LineP Transect (Pacific Ocean)	2 719	1 315	354	279	715
FTR_Aug2007	Feitsui Reservoir (Taiwan)	2 583	1 762	202	444	89
POV-LF26S	LineP Transect (Pacific Ocean)	2 504	1 641	284	269	233
POV-LJ26S	LineP Transect (Pacific Ocean)	2 414	1 414	686	148	85
POV-LJ4S	LineP Transect (Pacific Ocean)	2 410	1 813	279	133	103
POV-M5OD	MBARI transect (Pacific Ocean)	2 393	1 268	229	427	400
POV-M6O1K	MBARI transect (Pacific Ocean)	2 303	1 146	321	185	601
POV-LJ12O	LineP Transect (Pacific Ocean)	2 149	1 157	279	179	462
FTR_Jan2008	Feitsui Reservoir (Taiwan)	2 144	1 711	130	179	48
POV-M4OS	MBARI transect (Pacific Ocean)	1 587	884	60	364	255
POV-LF26A	LineP Transect (Pacific Ocean)	1 330	739	160	102	263
POV-M7O4K	MBARI transect (Pacific Ocean)	1 263	626	181	100	322
POV-GDS	Great Barrier Reef (Pacific Ocean)	1 088	525	79	288	169
SRR014589_Potable_DNA	Potable Water pond (Florida, USA)	1 082	963	49	27	34
POV-LF26O	LineP Transect (Pacific Ocean)	1 044	558	158	93	204
POV-LJ4O	LineP Transect (Pacific Ocean)	997	473	146	93	253
FTR_Aug2008	Feitsui Reservoir (Taiwan)	923	577	73	154	48
POV-GFS	Great Barrier Reef (Pacific Ocean)	915	483	59	209	151
POV-LJ26D	LineP Transect (Pacific Ocean)	826	598	87	71	45
POV-LJ12A	LineP Transect (Pacific Ocean)	807	452	124	70	123
POV-LJ26O	LineP Transect (Pacific Ocean)	804	493	116	71	104
POV-LJ4D	LineP Transect (Pacific Ocean)	801	421	97	56	201
POV-LF26D	LineP Transect (Pacific Ocean)	765	416	118	60	151
East_Lake_Wuhan_China_Aug2009	East Lake (Wuhan, China)	636	406	110	64	21
POV-LJ12D	LineP Transect (Pacific Ocean)	630	384	76	88	66
FTR_Jul2008	Feitsui Reservoir (Taiwan)	593	250	51	178	45
POV-LA26D	LineP Transect (Pacific Ocean)	592	341	86	42	88
POV-LA26O	LineP Transect (Pacific Ocean)	443	257	38	72	57
SRR014584_RW_Effluent_DNA	Reclaimed Water pond (Florida, USA)	410	263	34	41	52
POV-LA26A	LineP Transect (Pacific Ocean)	403	202	56	35	88
POV-M3MD	MBARI transect (Pacific Ocean)	386	239	46	56	33
JCVI_SMPL_1103283000058_mv858	Chesapeake Bay (Atlantic Ocean)	350	227	51	40	24
SRR014586_RW_Nursery_DNA	Reclaimed Water pond (Florida, USA)	261	161	28	37	28
Antarctic_Summer	Lake Limnopolar (Antarctica)	198	80	17	89	2
Antarctic_Spring	Lake Limnopolar (Antarctica)	107	30	12	64	0

Table S2. **Coverage of Far-T4 contigs in previously published viromes.** Only viromes with more than 100 hits are reported. Freshwater and seawater viromes are highlighted in green and blue respectively.
